# Isolation and characterization of *Schleiferilactobacillus harbinensis* GX0002947 from naturally fermented sour porridge and its application in cereal fermentation

**DOI:** 10.3389/fmicb.2025.1563733

**Published:** 2025-03-31

**Authors:** Guofang Liu, Yuanyuan Man, Hongmei Tian, Liuyan Wu, Qiao Li, Mingguo Jiang, Jin Dou, Huizhao Su

**Affiliations:** ^1^Guangxi Key Laboratory for Polysaccharide Materials and Modifications, School of Marine Sciences and Biotechnology, Guangxi Minzu University, Nanning, Guangxi, China; ^2^Sichuan Winshare Vocational College, Chengdu, Sichuan, China; ^3^The First Affiliated Hospital of Guangxi Medical University, Nanning, Guangxi, China; ^4^Guangxi Key Laboratory of Immunology and Metabolism for Liver Diseases, Nanning, Guangxi, China

**Keywords:** sour porridge, lactic acid bacteria, *Schleiferilactobacillus harbinensis* GX0002947, community differences, metabolomics

## Abstract

Sour porridge, a fermented food from the Guangxi Zhuang Autonomous Region of China, contains an abundance of lactic acid bacteria and has high nutritional value. In this study, a strain of *Schleiferilactobacillus harbinensis* GX0002947 was isolated from naturally fermented sour porridge from Fusui County, Chongzuo City, Guangxi Zhuang Autonomous Region of China. The strain was highly effective in the fermentation of sour porridge. It was found that strain *S. harbinensis* GX0002947 showed good acid and bile salt resistance at pH 3.5, bile salt concentration of 0.3 g/100 mL, in artificial gastrointestinal fluids, and the bacterial population density was greater than 10^6^ CFU/mL. The fermentation broth and culture supernatant of strain *S. harbinensis* GX0002947 showed effective antibacterial activity against the foodborne pathogens *Escherichia coli*, *Staphylococcus aureus*, and *Bacillus cereus*. The optimum fermentation process for sour porridge was found to consist of a fermentation temperature of 37°C, inoculation dose of 12.5%, and fermentation time of 96 h, resulting in a total protein content of 397.33 μg/mL and a total amino acid content of 629.63 μmol/mL in the sour porridge. In addition, the community diversity of fermented sour porridge was explored by high-throughput Illumina sequencing. The results showed that fermentation of sour porridge by *S. harbinensis* GX0002947 resulted in the formation of a unique microbial community. Metabolites were compared between sour porridge fermented by strain *S. harbinensis* GX0002947 and naturally fermented sour porridge and were analyzed by LC–MS. This identified 24 differential metabolites which primarily included amino acids, carbohydrates, and lipids, suggesting that the associated pathways played a key role in the fermentation of sour porridge by *S. harbinensis* GX0002947. In conclusion, this study used inoculation of lactic acid bacteria for the fermentation of sour porridge, and assessed differences in microbial community structure and metabolites after inoculation with *S. harbinensis* GX0002947. These findings provided a theoretical basis and technical support for sour porridge production.

## 1 Introduction

Sour porridge, also known as sour rice soup or sour lees, is a fermented cereal that is popular in the Guangxi Zhuang Autonomous Region, Shanxi Province, and Inner Mongolia Autonomous Region of China due to its sour taste, especially in summer, and digestive benefits (Zhang and Xia, 2020). In the Inner Mongolia Autonomous Region and the northwestern regions of Shanxi Province, minced rice, rice, and millet are typically used as the raw materials for sour porridge fermentation, during which the raw materials are placed in clean glass or ceramic jars and allowed to ferment naturally for 1 to 2 days. In contrast, in the Guangxi Zhuang Autonomous Region, sour porridge is made from cold rice or chilled rice porridge with natural fermentation in clay pots for 7 to 8 days (Ren et al., 2021). Microorganisms play crucial roles in the fermentation process, with various metabolites giving sour porridge its unique flavor.

Lactic Acid Bacteria (LAB) produce large amounts of lactic acid from carbohydrates such as sugar (Tian et al., 2021), and are widely used in various fermented products, such as fermented cereal, vegetables, and dairy products (Obioha et al., 2021). LAB were first discovered in the early 19th century, and numerous studies have analyzed their species and physiological and biochemical properties. To date, more than 80 genera and 700 species have been discovered, most of which are Gram-positive bacteria and generally do not produce spores (Klein et al., 1998; Singhvi et al., 2018; Alves De Oliveira et al., 2019). The most common LAB genera include *Lactobacillus*, *Streptococcus*, *Sporolactobacillus*, and *Leuconostoc* (Zotta et al., 2017; Milani et al., 2018; Zheng et al., 2020; Aslam et al., 2022), which have important roles in antibacterial activities, regulation of lipid metabolism, the intestinal flora, immunomodulation, and cholesterol reduction (Kouadri Boudjelthia et al., 2023; Rossi, 2023; Wang et al., 2023).

LAB are commonly used in the production of fermented cereal products such as breads, cakes, steamed bread, and cereal beverages. Fermentation reduces the digestibility of cereal starch while enhancing protein digestion and absorption, increasing the contents of dietary fiber and vitamins, and enhancing mineral bioavailability, thus improving the nutritional value of cereals (Verni et al., 2019). *L. fermentum*, *L. pentosus*, *L. casei*, *L. coryniformis*, *L. brevis*, *L. rossiae*, *L. reuteri*, and other *Lactobacillus* species are often used in fermented cereal products. Various organic acids, vitamins, alcohol, and flavor components are produced during the fermentation process, resulting in a wide variety of flavors in fermented products (Chen et al., 2020).

Due to the use of open fermentation processes, especially when making fermented sour porridge in a traditional family environment, bacterial contamination and spoilage can occur, leading to poor results. As probiotic bacteria, LAB produce lactic acid and other factors that can alter the food environment, inhibiting the growth and reproduction of harmful microorganisms and reduce food spoilage and deterioration, thus improving the safety and stability of the fermented food, extending the shelf life of the food (Axel et al., 2016). The metabolites produced by LAB have a wide range of biological activities that can promote the synthesis of various small molecules such as vitamins, amino acids, and other nutrients, enhancing the nutritional value of sour porridge (Hadj Saadoun et al., 2021). LAB fermentation is known to produce lactic acid, acetic acid, propionic acid, and other organic acids, all of which reduce the pH of the food, increase the sourness, and thus improve the overall taste of the sour porridge (Sneh et al., 2022). In addition, LAB can also produce a number of volatile compounds that contribute specific flavors to the porridge, improving the quality and attractiveness of the food.

Sour porridge has high nutritional value and a variety of beneficial effects. However, traditional sour porridge is still typically produced in family settings, which vary according to different geographical and climatic regions; this lack of standardization adversely affects the success of fermentation and cannot guarantee the quality of the porridge. The use of artificial inoculation of the microorganisms responsible for fermentation will thus ensure the consistent production of high-quality fermented sour porridge. However, although the use of LAB for the fermentation of sour porridge has many potential advantages, research on this topic is limited and the effects of different LAB species on sour porridge production remain unknown. The isolation and identification of LAB from sour porridge products and enriching current knowledge of these bacteria will improve the fermentation process, ensure the stability of quality, enrich the taste, and improve the nutritional value of the sour porridge, potentially leading to the industrial production of sour porridge.

## 2 Materials and methods

### 2.1 Strain and culture media

*Schleiferilactobacillus harbinensis* GX0002947 was isolated from naturally fermented sour porridge. The samples of sour porridge were collected from the houses of five farmers in Fusui County, Chongzuo City, Guangxi Zhuang Autonomous Region, China (107°3ˊE, 22°11ˊN). Three foodborne pathogens, *Escherichia coli*, *Bacillus cereus*, and *Staphylococcus aureus* were obtained from the Key Laboratory of Polysaccharide Materials and Modification, Guangxi Minzu University, Guangxi Zhuang Autonomous Region, China. *S. harbinensis* GX0002947 was cultured in MRS Medium. Three foodborne pathogens were cultured in LB medium.

### 2.2 Isolation and identification of strain GX0002947

The fermented sour porridge samples were diluted to suitable concentrations, streaked on CaCO_3_-MRS medium, and cultured at 37°C for 48 h. Colonies with obvious calcium-soluble rings were selected and single colonies with the same morphology were obtained after several purifications.

The 16S rRNA gene sequence was analyzed to identify strain GX0002947. Genomic DNA was extracted from strain GX0002947 and amplified by PCR using the universal primers 27F (5’-AGAGTTTGATCMTGGCTCAG-3′) and 1492R (5′-GGTTACCTTGTTACGACTT-3′) for the 16S rRNA gene. The PCR products were sent to Shanghai Sangon (Shanghai, China) for sequencing. The 16S rRNA gene sequences were compared using BLAST on the EzBioCoud website^1^, and a phylogenetic tree was constructed using MEGA 11 software.

The metabolic experiments of sugar (alcohol): a complete set of lactic acid bacteria biochemical identification tube SHBG13 (Qingdao Haibo Biotechnology Co., Ltd., China) was used for experiments. The ampoules were first taken out of the packaging box, and then were folded in the opposite direction of the easy fold point with a grinding wheel, inserted into the test tube rack. Objective strains were purified by MRS plate in advance. The strains that had been purified and cultured were inoculated with an inoculation loop and inoculated in the biochemical tube where the experiment was required, sealed after inoculation, and incubated at 37°C for 48 h. According to the kit instructions, the color results of the biochemical tube can be divided into positive results and negative results. Aesulin appears dark or yellow is positive, while purple or grayish is negative. Cellobiose, Maltose, Mannitol, Salicin, Sorbitol, Sucrose, Raffinose, Inulin and Lactose appear yellow is positive, while purple or grayish purple is negative. 1% Sodium hippurate appears dark purple is positive, while lavender or colorless is negative.

A SUPRA 55 Sapphire scanning electron microscope (SEM, Carl Zeiss, Germany) was used for the evaluation of the morphology of the strains. Gram-positive and Gram-negative bacteria were identified using a Gram-bacterial staining kit (Guangdong Huankai Microbial Technology Co., Ltd., China). The target strains were processed by crystal violet staining, alcohol decolorization, and sandy yellow re-staining according to the provided directions, and examined under a light microscope (Sgibnev and Kremleva, 2017).

### 2.3 Detection of probiotic properties

#### 2.3.1 Acid tolerance test

The pH of the MRS medium was adjusted to 2.5, 3.5, or 4.5 using hydrochloric acid, followed by sterilization at 121°C for 20 min. The strain was inoculated at 1% inoculation dose (OD_600_ = 1.0) in the different media and was cultured at 37°C. 100 μL of the bacterial suspension were collected at 0 and 3 h, inoculated on CaCO_3_-MRS agar plates, and cultured at 37°C for 48 h. The number of viable bacteria was recorded (Yang E. et al., 2018; Yang et al., 2024). The survival rate was calculated as follows:


$$\mathrm{Survival rate}\left(\%\right)=\frac{N_{1}}{N_{0}}\times 100\%$$

Where: N_1_, the number of viable bacteria after incubation for 3 h, CFU/mL; N_0_, the number of viable bacteria after incubation for 0 h, CFU/mL.

#### 2.3.2 Bile salt tolerance test

MRS broth media containing bile salts at concentrations of 0.03, 0.3, and 0.5 g/100 mL were prepared. MRS broth medium without bile salts was used as the control. The strain was inoculated at a 1% inoculation rate (OD_600_ = 1.0) into the above media, and was cultured at 37°C. 100 μL of the bacterial suspension were collected at 0 and 3 h, inoculated on CaCO_3_-MRS agar, and cultured at 37°C for 48 h (Ko et al., 2023; Santos et al., 2023). The number of viable bacteria was recorded, and the survival rate was calculated as above.

#### 2.3.3 Artificial gastrointestinal fluid tolerance test

Artificial gastric intestinal fluids were configured according to the Chinese Pharmacopoeia. The strain was grown in MRS liquid medium until the OD_600_ was 1.0 after which it was inoculated at a 1% inoculation rate in MRS liquid medium and cultured at 37°C for 48 h. The culture was centrifuged, the supernatant discarded, and the same volume of sterile saline was added to suspend the pelleted bacteria. The bacterial suspension was mixed with artificial gastric and intestinal fluid at a ratio of 1:9, followed by incubation at 37°C. Aliquots were collected at 0 and 3 h and serially diluted. 1 mL of the bacterial suspension was streaked on MRS agar, cultured at 37°C for 48 h, and the number of viable bacteria was recorded. The survival rate was calculated as above.

#### 2.3.4 Antibacterial activity test

The antibacterial activity of the target strain was evaluated using the Oxford Cup method (Dinçer and Kıvanç, 2021). Three common foodborne pathogens, *Escherichia coli*, *Bacillus cereus*, and *Staphylococcus aureus* were used as indicator strains to determine the antibacterial activity of the target strain. The three indicator strains and strain GX0002947 were inoculated into LB broth medium or MRS broth medium and incubated at 37°C with shaking until the OD_600_ was 1.0. 100 μL suspensions of the three indicator bacteria were streaked on LB medium, after which a sterilized Oxford Cup was placed on the surface of the medium and pressed for fixation. 200 μL of the fermentation broth and cell-free fermentation supernatant of strain GX0002947 were absorbed and cultured in the Oxford Cup at 37°C for 48 h. The diameter of the inhibition circle was measured using vernier calipers, and the antibacterial activity of the target strain was determined.

### 2.4 Optimization of fermentation conditions for sour porridge

The fermentation process consisted of: rice → pretreatment (washing) → addition of water (water-to-rice ratio of 5:1, v/w) → rice medium preparation → sterilization → bacterial suspension preparation → inoculation fermentation (natural fermentation with 2.0% equal volume of glucose) → finished product.

Strain GX0002497 was inoculated into the rice medium, and the fermentation time, inoculation dose, and fermentation temperature were optimized using the total protein concentration as the main index. For the optimization of fermentation time, strain GX0002947 was inoculated at a 10% inoculation rate in the rice medium and cultured at 37°C for varying times, namely, 0, 12, 24, 36, 48, 60, 72, 84, 96, 108, and 120 h. The total protein content of the sour porridge produced using different fermentation times was then determined. For optimization of the inoculation dose, strain GX0002947 was inoculated at 2.5, 5.0, 7.5, 10, 12.5, and 15% inoculation rates in rice medium and fermented at 37°C for 96 h. The total protein content of the fermented sour porridge produced with different inoculation amounts was determined. For the optimization of the fermentation temperature, strain GX0002947 was inoculated at a 12.5% inoculation rate in rice medium, and fermented at 28, 31, 34, 37, 40, or 43°C for 96 h. The total protein content of the sour porridge fermented at the different temperatures was then determined.

### 2.5 Analysis of the physical and chemical properties and nutrient composition of sour porridge

Determination of pH value: a pH meter was used to measure the pH value of naturally fermented and inoculated fermented sour porridge samples. The pH value was recorded, with three measurements performed in each case.

Titratable acid determination: the naturally fermented and inoculated fermented sour porridge samples were stirred evenly. First, ddH_2_O was added to each sample, followed by a few drops of phenolphthalein indicator. The samples were titrated with a 0.1 mol/L NaOH standard solution, with an endpoint when the solution was slightly red and did not fade for 30 s. The volume of NaOH consumed by the samples was recorded, with three parallel measurements performed for each sample. The titratable acid was calculated as follows:


$$X=\frac{c\times \left(V-V_{0}\right)\times 10.0}{\;m\times 0.1}$$

Where: X: acidity of the sample, °T; c: molar concentration of NaOH standard solution, mol/L; V: volume of NaOH used in the titration, mL; V_0_: volume of NaOH used in the blank, mL; 10.0: 10.0 g sample; m: mass of the sample, g; 0.1: concentration of NaOH as defined by acidity theory, mol/L.

Determination of total protein content: the total protein contents of naturally fermented and inoculated fermented sour porridge samples were determined using a Total Protein Content assay kit (Nanjing Jiancheng, China), with three replicates per sample.

Determination of total amino acid content: the total amino acid contents of naturally fermented and inoculated fermented sour porridge samples were determined using a Total Amino Acid Content Detection Kit (Nanjing Jiancheng, China), with three replicates per sample.

### 2.6 Analysis of microbial diversity and differential metabolites in naturally fermented and inoculated fermented sour porridge

#### 2.6.1 Analysis of microbial diversity in sour porridge

The sour porridge was fermented at 37°C for 96 h according to Materials and methods 2.4. After the fermentation was completed, 50.0 g of naturally fermented sour porridge and *S. harbinensis* GX0002947-inoculated fermented sour porridge were, respectively, weighed and placed into sterile centrifuge tubes in three parallels for each group. They were, respectively, named as the ZR group and the DD group for the convenience of subsequent data analysis.

Genomic DNA was extracted from the sour porridge samples and assessed using 1% agarose gel electrophoresis. PCR amplification of bacteria and fungi was performed using the primers 27F/1492R and ITS1/ITS4, respectively (Yang Z. Y. et al., 2018; Zhai et al., 2022; Ye and Lei, 2023). After quality selection, samples were sent to Shanghai Majorbio Bio-pharm Technology Co., Ltd. (Shanghai, China) for metagenomic sequencing.

The raw reads were filtered for quality by removal of low-quality reads to obtain clean reads which were then spliced by Megahit software to obtain the spliced contigs. The contigs with the best splicing effects were selected. The open reading frames were predicted and CD-HIT software was used to cluster the predicted gene sequences. The longest gene in each cluster was selected as the representative sequence, resulting in the construction of a non-redundant gene set. Using a 95% similarity level, the sequences of the clean reads from each sample and the constructed non-redundant gene set were compared using SOA Paligner software, followed by the determination of abundance and annotation of species, analyzed abundance at the phylum, genus, and species levels. The diversity and abundance of bacterial community structure and the differences in the community structure composition between the two groups were compared, determining the *α* diversity and *β* diversity indices, respectively.

#### 2.6.2 Metabolomics analysis of fermented sour porridge using LC–MS

Sample preparation: the rice was fermented according to the sour porridge fermentation process in 2.4. After fermentation, the naturally fermented sour porridge and inoculated fermented sour porridge by strain GX0002947 were weighed 50.0 g into aseptic centrifuge tubes with three parallel sets in each group, and named comp 1 and comp 2, respectively for the convenience of subsequent data analysis. Then the naturally fermented sour porridge samples and artificially inoculated fermented sour porridge samples were sent to Shanghai Majorbio Bio-pharm Technology Co., Ltd. (Shanghai, China) for metabolomic sequencing.

The chromatographic conditions used were (Yang et al., 2021; Zhang et al., 2023): An ACQUITY UPLC HSS T3 column (100 mm 2.1 mm × 1.8 μm; Waters, Milford, United States) was used. The mobile phase A consisted of 95% water and 5% acetonitrile (containing 0.1% formic acid), and mobile phase B consisted of 47.5% acetonitrile, 47.5% isopropyl alcohol and 5% water (containing 0.1% formic acid). The injection volume was 3 μL, and the column temperature was 40°C.

The parameters used were: scanning range, 70–1,050 m/z; sheath gas flow rate, 50 arb; auxiliary gas flow rate, 13 arb; heating temperature, 425°C; capillary temperature, 325°C; spray voltage (positive mode) 3,500 V; spray voltage (negative mode), −3,500 V; S-Lens voltage 50 V; collision energies 20, 40, 60%; resolution 60,000; full MS resolution, 7,500 MS^2^.

### 2.7 Statistical analysis

Data were organized and calculated in Excel, while SPSS 22.0 was used for analysis. The data are expressed as mean ± standard deviation, and were compared using one-way analysis of variance (ANOVA). The results were visualized using GraphPad Prism 9, and the correlation network was mapped using the Majorbio Cloud platform.^2^

The LC–MS data were normalized with the simulation of missing values to facilitate the screening and analysis of target differential metabolites. The data were preprocessed on the Majorbio Cloud platform, and the processed data were modeled and analyzed by orthogonal partial least squares discriminant analysis (OPLS-DA). Differential metabolites between the two sample groups were identified using the criteria of *p* < 0.05, fold change (FC) ≥ 1.5, and the importance of variable projection (VIP) ≥ 1. Metabolites were identified and analyzed using KEGG Compound Classification R Language 2017.05.01 and KEGG Pathway Enrichment Python v1.0.0.

## 3 Results and discussion

### 3.1 Isolation and identification of LAB

**Figure 1:**
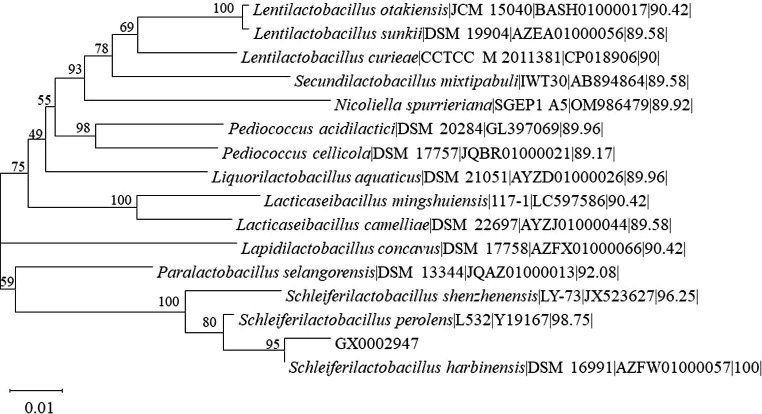
Phylogenetic tree of 16S rRNA gene sequences from GX0002947 and other lactic acid bacteria.

In this study, a total of 75 strains of five *Lactobacillus* species were isolated from 7 samples of sour porridge taken from Fusui County, Chongzuo City, Guangxi Zhuang Autonomous Region of China. Through morphology and 16S rRNA gene identification, BLAST comparison, and species classification, it was found that the strains in the sour porridge samples belonged primarily to the *Lactobacillus* (including *Lacticaseibacillus*, *Schleiferilactobacillus*) and *Bacillus* genera. Physiological and biochemical characterization revealed that most of all strains were negative for peroxidase. The phylogenetic tree showed that strain GX0002947 was clustered on the same branch as *S. harbinensis* DSM16991, showing 100% similarity (Figure 1), and it was thus termed *S. harbinensis* GX0002497. According to the decomposition ability of different bacteria to various sugar alcohols and the metabolites produced, some can decompose a variety of sugars (alcohols), some can only decompose 1 ~ 2 kinds of sugars (alcohols), some decompose sugars (alcohols) can produce acid and gas, and some decomposing sugar (alcohol) produces only acid but not gas. Based on these characteristics, bacteria can be identified. Through the sugar alcohol utilization experiments, we found that strain *S. harbinensis* GX0002947 could use Mannitol, Salicin, Sorbitol, and 1% Sodium hippurate. However, strain *S. harbinensis* DSM16991 could not utilize these sugar alcohols (Supplementary Table S1). Morphological characterization revealed that the strain was a Gram-positive bacterium, with a rod-like structure on SEM (Supplementary Figure S1).

### 3.2 Probiotic characterization of the strain GX0002947

#### 3.2.1 Acid tolerance analysis

Sour porridge enters the human stomach through the esophagus and enters the stomach for 3 h at a pH range of 2.5–3.5 (Ma, 2024). Therefore, the pH values of 2.5, 3.5, and 4.5 were selected for analysis of acid tolerance of the strain *S. harbinensis* GX0002947. Strain GX0002947 was transferred to MRS broth medium with pH 2.5, 3.5, or 4.5 for 0 and 3 h, and the number of viable bacteria was counted. The results (Table 1) showed that the acid resistance was over 80%, with a survival rate of 81.08% at pH 2.5. The number of viable bacteria at pH 3.5 and pH 4.5 after 3 h was higher than 10^6^ CFU/mL and thus met the minimum standard of probiotics activity, indicating that strain GX0002497 had good acid resistance and could maintain high activity for a specific time in the acid conditions of the stomach. *S. harbinensis* GX0002947 could thus maintain a high survival rate under acidic conditions, which might be due to the presence of specific regulatory mechanisms or factors, enabling the strain to maintain its intracellular pH without being affected by environmental conditions. The ability of probiotics to survive at low pH and high bile salt environments is an important features of successful passage through the gastrointestinal tract (de Souza et al., 2019; Ramos et al., 2013).

**Table 1:** Acid resistance of *S. harbinensis* GX0002947.

Strain	pH	0 h Viable count (10^6^ CFU/mL)	3 h Viable count (10^6^ CFU/mL)	Survival rate (%)
GX0002947	2.5	0.37 ± 0.09	0.30 ± 0.08	81.08
	3.5	1.35 ± 0.07	1.90 ± 0.08	140.74
	4.5	1.50 ± 0.06	3.95 ± 0.07	263.33

The data in the table is the mean standard deviation of three repeated experiments.

#### 3.2.2 Bile salt tolerance analysis

The prerequisite for LAB as probiotics is their ability to survive and colonize in the intestines. After passage through the stomach, the LAB come into contact with bile salts in the intestines (the mass concentration of bile salt fluctuates from 0.03 to 0.3 g/100 mL). The food remains for between 1 and 4 h in the small intestine (Li et al., 2024). *S. harbinensis* GX0002947 was inoculated into MRS broth medium with bile salt concentrations of 0.03, 0.3, and 0.5 g/100 mL for 0 and 3 h, and its ability to tolerate bile salts was examined by counting the number of viable bacteria. As shown in Table 2, strain GX0002947 could survive for 3 h under all three bile salt concentrations, with survival rates of 134, 30.05, and 9.46%, respectively. This indicated that the higher concentrations of bile salts reduced the survival of the bacteria. This might be explained by the induction of an osmotic pressure gradient by higher bile salt concentrations between the interior and exterior of the bacterial cell, resulting in cell death. It was also possible that high concentrations of bile salts destroyed phospholipids and proteins in bacterial cell membranes, changed membrane permeability, and caused damage or death (Cai et al., 2022; Collins et al., 2023). Nevertheless, after 3 h of incubation at bile salt concentration of 0.03, and 0.3 g/100 mL, the number of viable bacteria was greater than 10^6^ CFU/mL, which was in line with the standard for probiotics. Therefore, *S. harbinensis* GX0002947 had an adequate tolerance for bile salts.

**Table 2:** Bile salt tolerance of *S. harbinensis* GX0002947.

Strain	Bile salt (g/100 mL)	0 h Viable count (10^6^ CFU/mL)	3 h Viable count (10^6^ CFU/mL)	Survival rate (%)
GX0002947	0.03	7.00 ± 0.09	9.38 ± 0.09	134
	0.3	3.76 ± 0.07	1.13 ± 0.06	30.05
	0.5	0.74 ± 0.08	0.07 ± 0.07	9.46

The data in the table is the mean standard deviation of three repeated experiments.

#### 3.2.3 Tolerance of artificial gastrointestinal fluids

LAB have a high intestinal adhesion rate and readily colonize in the intestinal tract. It can modulate the intestinal flora, prevent and treat diarrhea, prevent allergy, improve immunity, and exhibit anticancer activity (Björkstén, 2004; Hojsak et al., 2010; Liu et al., 2020; Osterlund et al., 2007; Riaz Rajoka et al., 2018). In recent year, scientists have begun to study probiotics in sour porridge, with the aim of evaluating the effect of microbes in sour porridge on human intestinal health (Nout et al., 1989; Tetteh et al., 2004). Although the acid and bile salt tolerance experiments simulated the human gastrointestinal environment through high acid and bile salt concentrations, the pepsin and trypsin present in the gastrointestinal environment *in vivo* will hydrolyze microbial proteins and even inhibit or kill the microorganisms. Gastric acid and pepsin are the primary components of human gastric juice, and probiotics entering the human stomach can only function optimally if they can colonize the lower part of the intestine (Liebeke et al., 2012; Seo et al., 2020). The results of these analyses are shown in Tables 3, 4. After treatment of *S. harbinensis* GX0002947 with artificial gastric juice for 3 h, the survival rate was 67.74%. In the simulated intestinal environment, the survival rate was 65.65%, which was greater than 50% and the number of viable bacteria was still higher than 10^6^ CFU/mL, indicating that *S. harbinensis* GX0002947 could tolerate artificial gastrointestinal fluids, consistent with the above acid tolerance results.

**Table 3:** Tolerance of artificial gastric juice of *S. harbinensis* GX0002947.

Strain	0 h Viable count (10^6^ CFU/mL)	3 h Viable count (10^6^ CFU/mL)	Survival rate (%)
GX0002947	0.93 ± 0.06	0.63 ± 0.08	67.74

The data in the table is the mean standard deviation of three repeated experiments.

**Table 4:** Tolerance of artificial intestinal juice of *S. harbinensis* GX0002947.

Strain	0 h Viable count (106 CFU/mL)	3 h Viable count (106 CFU/mL)	Survival rate (%)
GX0002947	5.59 ± 0.07	3.67 ± 0.05	65.65

The data in the table is the mean standard deviation of three repeated experiments.

#### 3.2.4 Antibacterial activity analysis

**Figure 2:**
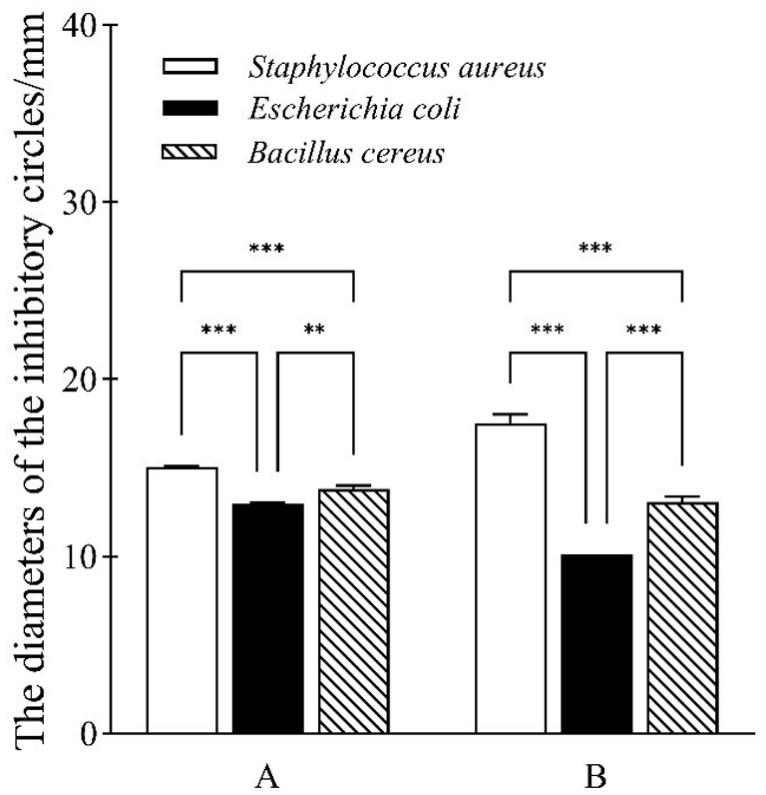
Inhibitory effect of fermentation broth and supernatant of *S. harbinensis* GX0002947 on three foodborne pathogens. **(A)** Fermentation broth of strain GX0002947; **(B)** The supernatant of strain GX0002947. Data represent mean ± standard deviation from three replicates, ***, *p* < 0.001; **, *p* < 0.01.

Small numbers of pathogenic food bacteria such as *Bacillus cereus* were isolated from sour porridge samples in this study, indicating the presence of mold and bacterial pathogens in traditionally fermented sour porridge, resulting in its uneven quality, while LAB can secrete a variety of antibacterial substances during the fermentation process, including organic acids, bacteriocin, and hydrogen peroxide (Chen et al., 2021). In this study, three common foodborne pathogens, *Staphylococcus aureus*, *Bacillus cereus*, and *Escherichia coli*, were used as indicator bacteria to investigate the antibacterial properties of *S. harbinensis* GX0002947. As shown in Figure 2, the fermentation broth and culture supernatant of strain GX0002947 differed in their inhibitory effects on the indicator strains, although both could inhibit the indicator bacteria. Among them, the inhibitory effect of *S. harbinensis* GX0002947 fermentation broth on the three indicator bacteria was ranked in the order of *Staphylococcus aureus* > *Bacillus cereus* > *Escherichia coli*, with respective inhibitory circle diameters of 15.0, 13.8, 13.0 mm. The ranking of the inhibitory effects of the strain GX0002947 culture supernatant on the three indicator bacteria was the same as that of the fermentation broth, although the diameters of the inhibitory circles differed, at 17.5, 13.1, and 10.1 mm, respectively. In summary, the culture supernatant and fermentation broth of *S. harbinensis* GX0002947 had different inhibitory effects on the three foodborne pathogens. It could be seen that *S. harbinensis* GX0002947 might secrete some antibacterial substances to inhibit the growth and reproduction of pathogens.

### 3.3 Optimization of fermentation conditions of strain GX0002947

The optimal fermentation conditions for sour porridge were evaluated to maximize the value of the final product. Thus, the differences between fermented sour porridge with *S. harbinensis* GX0002947 and naturally fermented sour porridge were assessed. The fermentation time, inoculation dose, and fermentation temperature have significant effects on the nutritional value of sour porridge and the success of the fermentation process (Fan et al., 2017; Ren et al., 2022; Schwarz et al., 2022). The study found that soluble proteins increased 10-fold during millet fermentation, which was attributed to microbial enzyme activity and protein hydrolysis (Sripriya et al., 1997). In the process of soybean fermentation, soluble nitrogen increased by 47% in 6 to 9 h of fermentation (Sarkar and Tamang, 1995), and the increase of soluble nitrogen and free amino acids was attributed to the hydrolysis of soybean protein (Ekachai, 2015). Therefore, in this study, the fermentation time, inoculation dose, and fermentation temperature were used as single factors, with the total protein concentration used as the evaluation index, in a single-factor experiment on strain GX0002947 fermentation of sour porridge.

**Figure 3:**
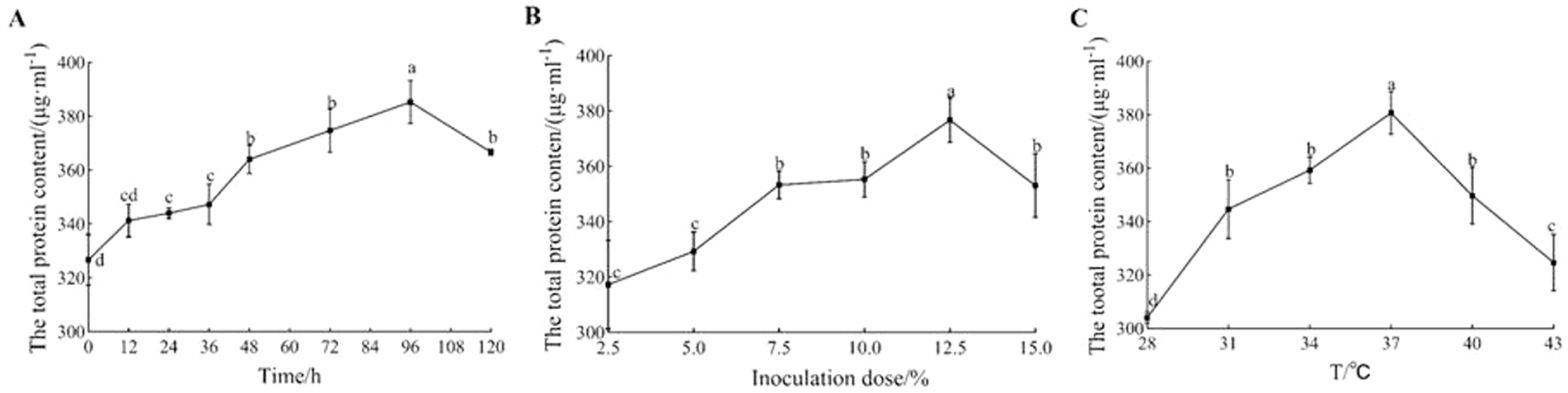
Optimization of fermentation conditions of strain GX0002947. **(A)** Optimization of fermentation time; **(B)** Optimization of inoculation dose; **(C)** Optimization of fermentation temperature. Data represent mean ± standard deviation from three replicates. In the same picture, any different lowercase letter indicates a significant difference (*p* < 0.05), and any same lowercase letter indicates a non-significant difference (*p* > 0.05).

The fermentation time has a direct effect on the quality of sour porridge products. As shown in Figure 3A, the total protein concentration of sour porridge fermented by strain GX0002947 increased first and then decreased with the extension of the fermentation time; the protein concentration was 385.33 μg/mL after fermentation for 96 h, which was significantly higher than that at other fermentation times (*p* < 0.05). At this time point, the total protein concentration of the sour porridge was highest and the flavor was best. Too long or too short a fermentation time affected the success of the fermentation, with reduced protein contents and a loss of nutrients.

The inoculation dose has a marked influence on the fermentation process of sour porridge. Inoculation doses that are too high or too low will adversely affect the fermentation process. As shown in Figure 3B, the total protein concentration in sour porridge fermented by strain GX0002947 showed a similar trend of initial increase followed by a decrease as the inoculation dose increased. At a dose of 12.5%, the total protein concentration of the sour porridge was 376.67 μg/mL, which was significantly higher than those seen with other inocula (*p* < 0.05). After fermentation, the total protein concentration was highest with the best flavor. In general, if the inoculation dose is too low, fermentation will be retarded, prolonging the production cycle, which is not suitable for industrial production. On the other hand, if the inoculation dose is too high, it will increase the production cost, and LAB will also compete for nutrients for their own reproduction, reducing their growth and metabolic functions and further contributing to reductions in the total protein concentration.

The fermentation temperature affects the growth and reproduction of the strains, and thus affects the fermentation process of sour porridge. As shown in Figure 3C, the total protein concentration of sour porridge fermented by strain GX0002947 also showed a trend of first increasing and then decreasing as the temperature increased. At 37°C, the total protein content was 380.67 μg/mL, which was significantly higher than that at the other fermentation temperatures (*p* < 0.05). The sour porridge had the highest total protein content and the best flavor at this fermentation temperature. Both high and low temperatures will affect the growth and metabolic functions of fermentation strains, leading to reduce total protein contents after fermentation and resulting in nutritional loss.

According to the optimal fermentation conditions, *S. harbinensis* GX0002947 was inoculated for sour porridge fermentation. It was found (Table 5) that the pH value of sour porridge fermented by strain GX0002947 was 2.92, which was lower than naturally fermented sour porridge (*p* < 0.05). Titratable acid of sour porridge fermented by strain GX0002947 was 3.02 °T, which was significantly higher than that of naturally fermented sour porridge (*p* < 0.05). It can be inferred that adding strain GX0002947 for fermentation may produce a large amount of organic acids, resulting in the change of pH and titratable acid of sour porridge, and the growth and reproduction of harmful bacteria are inhibited under low pH and high acidity, thus improving the success rate of sour porridge fermentation. The total protein content and total amino acid content of *S. harbinensis* GX0002947 fermented sour porridge were 397.33 μg/mL and 629.63 μmol/mL, respectively, which were much higher than naturally fermented sour porridge samples (*p* < 0.05). It can be inferred when artificially inoculated microorganisms fermented sour porridge, microorganisms may secrete a large amount of protein, which increases the total protein content, and can metabolize and produce free amino acids, further improving the nutritional value of sour porridge.

**Table 5:** Changes of physicochemical properties and nutritional composition of naturally fermented and *S. harbinensis* GX0002947 fermented sour porridge.

Sour porridge samples	pH	Titration acidity (°T)	Total protein content (μg/mL)	Total amino acid content (μmoL/mL)
Naturally fermented	5.70 ± 0.06	0.31 ± 0.01	299.40 ± 2.00	99.01 ± 11.33
Strain GX0002947 fermented	2.92 ± 0.09	3.02 ± 0.29	397.33 ± 3.06	629.63 ± 32.08

The data in the table is the mean standard deviation of three repeated experiments.

### 3.4 Changes in microbial community structure during sour porridge fermentation

#### 3.4.1 Sequence richness and diversity analysis

The Chao1 and Shannon indices are commonly used to measure the total number and diversity of bacterial communities (Cherdyntseva et al., 2016; Alameri et al., 2022). In this study, the community composition of each sample was first classified according to the phylum, class, order, family, and genus taxonomic levels, as well as the species diversity index. As shown in Supplementary Table S2, a total of 611 microorganisms were identified from the three samples of naturally fermented sour porridge (ZR-1, ZR-2, and ZR-3) and three samples of sour porridge fermented by *S. harbinensis* GX0002947 (DD-1, DD-2, and DD-3). These microorganisms belonged to 5 phyla, 13 classes, 15 orders, 15 families, and 18 genera.

Larger Chao1 or Sobs indices indicate greater species numbers and richness of microbial communities, while large values of the Simpson index indicate lower community diversity (Marco et al., 2020). The alpha diversity indices of naturally fermented sour porridge and sour porridge samples fermented by strain *S. harbinensis* GX0002947 are shown in Table 6. Compared with the naturally fermented samples, the Simpson index was higher in the samples fermented by *S. harbinensis* GX0002947, while the value of the Shannon index was smaller, indicating that the microbial community diversity of the strain-fermented samples was lower relative to that of the naturally fermented samples. Compared with the natural fermentation samples, the Chao1 and Sobs indices were lower for porridge fermented by *S. harbinensis* GX0002947, indicating lower species abundance and also that the experimental fermentation process had not been contaminated by miscellaneous bacteria.

**Table 6:** Alpha diversity index table of naturally fermented sour porridge and *S. harbinensis* GX0002947 fermented sour porridge.

Type of fermentation	Simpson’s index	Shannon index	Sobs index	Chao1 index
Naturally fermented	0.9979 ± 0.0005	0.0084 ± 0.0020	3.6700 ± 0.5773	3.6700 ± 0.5773
Strain GX0002947 fermented	0.9999 ± 0.0001	0.0002 ± 0.0001	3.3300 ± 0.5773	3.3300 ± 0.5773

#### 3.4.2 Analysis of community structures in sour porridge

**Figure 4:**
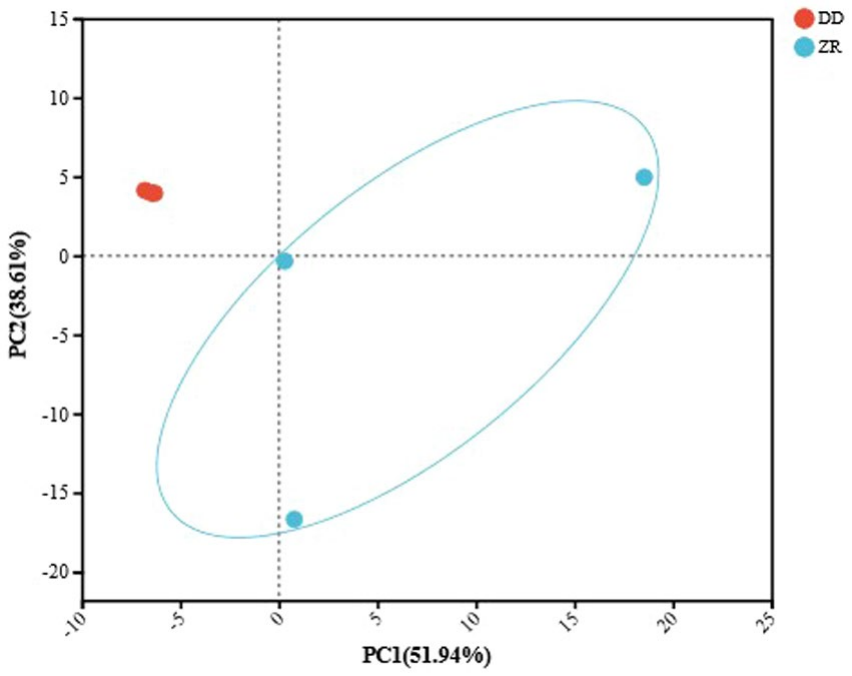
PCA analysis of naturally fermented sour porridge and *S. harbinensis* GX0002947-inoculated fermented sour porridge at species level.

Species differences between microbiomes are often represented by beta diversity analysis. Principal component analysis (PCA) enables the visualization of differences and distances according to their composition, with shorter distances indicating more similar species compositions (Zhong et al., 2020; Qin et al., 2024). As shown in Figure 4, the top three principal components contributed 51.94, 38.61, and 8.81%, respectively, and the cumulative contribution rate was 99.38%, indicating that PCA could retain the main features of the original data and meet the requirements of the analysis. The PCA results showed that the three samples fermented by *S. harbinensis* GX0002947 were closer together and could be clustered into one category. In contrast, the three samples of naturally fermented sour porridge were more distant, indicating greater differences in the species composition of the microorganisms. It could be seen that the internal community and species composition of artificially inoculated fermented sour porridge were more stable, while naturally fermented sour porridge was susceptible to interference by environmental and other factors during the fermentation process, resulting in differences in the community and species composition of the fermented sour porridge products, with little guarantee of the quality of the products.

**Figure 5:**
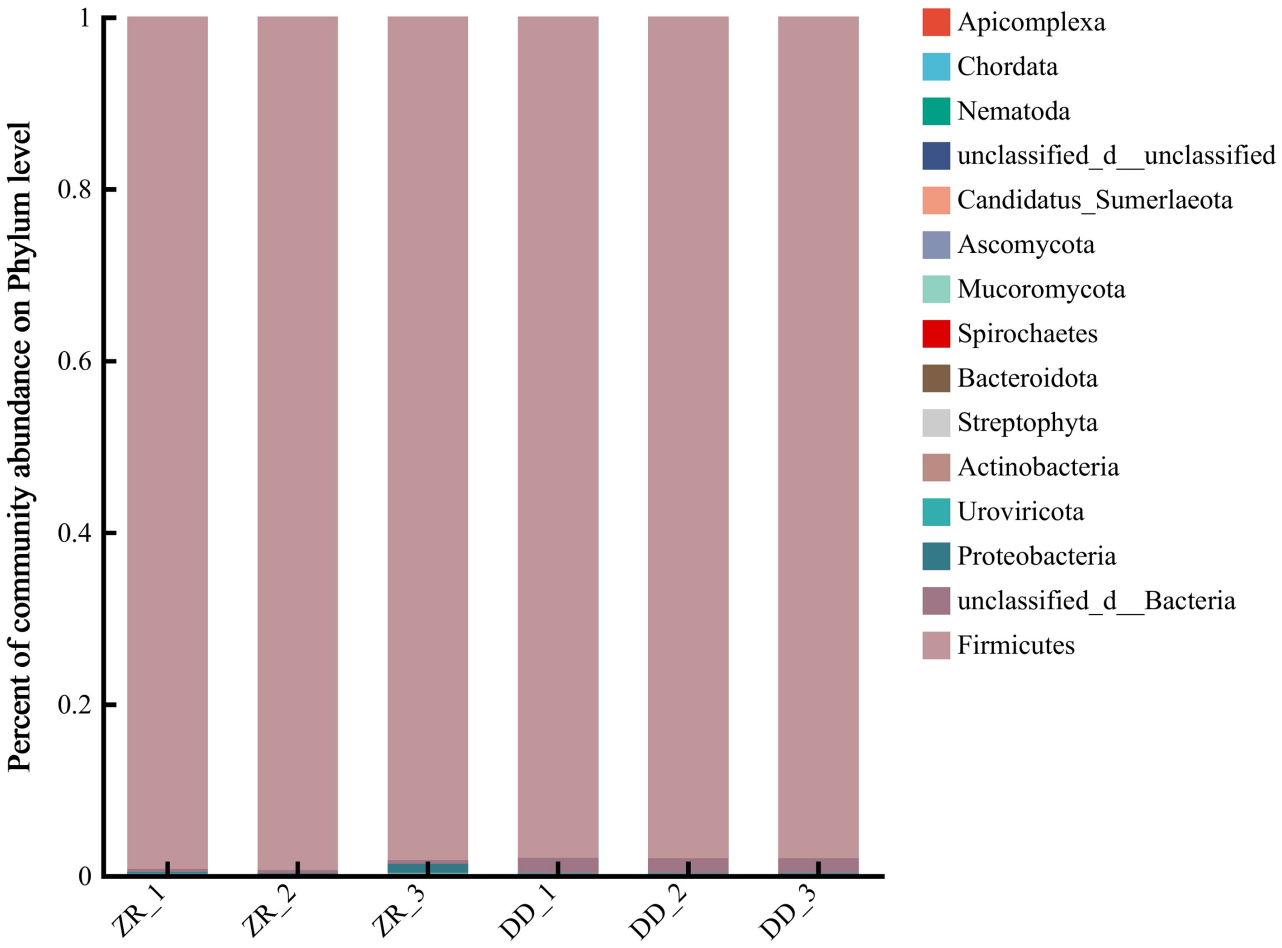
Relative abundance of horizontal flora in phylum level of naturally fermented sour porridge and *S. harbinensis* GX0002947-inoculated fermented sour porridge.

The community compositions at the phylum level are shown in Figure 5 and Supplementary Table S3. There were three dominant phyla in the three naturally fermented samples, namely, Firmicutes, Proteobacteria, and Actinobacteria, respectively. More than 98.2% of the bacterial genes were from Firmicutes, and most of the remaining genes were from Proteobacteria. The three *S. harbinensis* GX0002947-inoculated fermented sour porridge samples contained mostly Firmicutes, contributing over 98.0% of the genes. It can thus be seen that most of the bacteria in the two groups of fermented sour porridge samples were from Firmicutes, while the naturally fermented sour porridge samples also contained Proteobacteria. There were thus differences in the composition of bacterial and fungal phyla between the different sour porridge samples.

**Figure 6:**
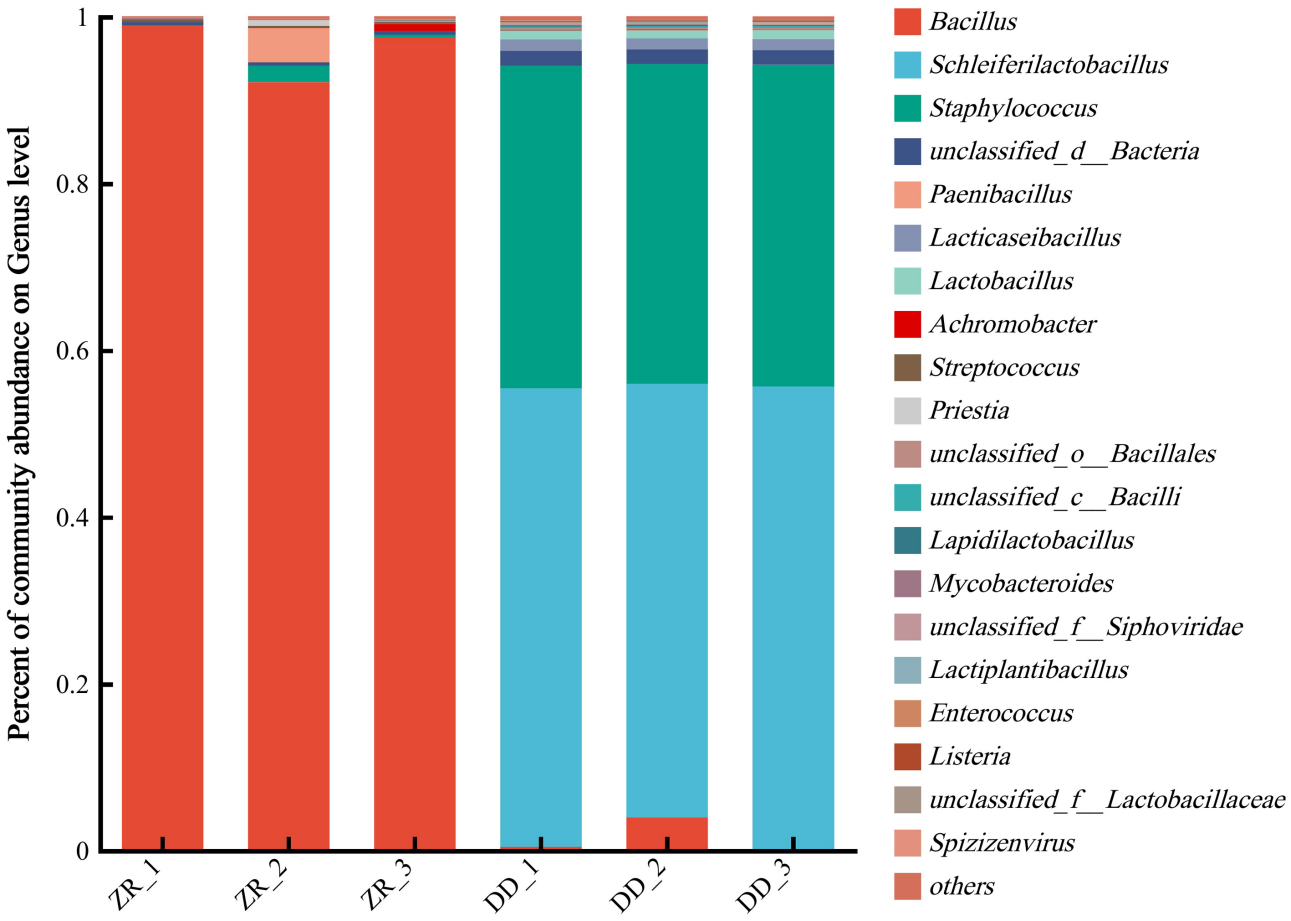
Relative abundance of horizontal flora in genus level of naturally fermented sour porridge and *S. harbinensis* GX0002947-inoculated fermented sour porridge.

The community compositions were further analyzed at the genus level. As shown in Figure 6 and Supplementary Table S4, a total of 18 genera were identified in the two groups of fermented sour porridge samples, with a greater abundance of genera in the naturally fermented sour porridge samples than in the inoculated fermented samples. The bacteria in three samples of naturally fermented sour porridge included *Bacillus*, *Paenibacillus*, *Staphylococcus*, *Priestia*, *Streptococcus*, and among others. Among them, *Bacillus* was the most abundant, composed mostly of *Bacillus velezensis, Bacillus subtilis* and *Bacillus amyloliquefaciens*. The sour porridge samples fermented by *S. harbinensis* GX0002947 contained primarily *Schleiferilactobacillus*, *Staphylococcus, Bacillus*, *Lacticseibacillus*, and *Lactobacillus*. Among them, *S. harbinensis* was the most abundant and was the dominant strain. The community structures differed markedly at the genus level between the two groups of fermented sour porridge samples. The inoculated fermented porridge samples showed enrichment with large numbers of *Staphylococcus* bacteria, suggesting that there might be collaborative fermentation between the two genera of bacteria during the sour porridge fermentation process. In the ZR-1, ZR-2, and ZR-3 samples of naturally fermented sour porridge, the proportions of sequences that could not be classified in terms of genus were 0.45, 0.39, and 0.38%, respectively, while the proportions in the artificially inoculated fermented sour porridge samples DD-1, DD-2, and DD-3 were 1.77, 1.76, and 1.76%, respectively. This indicated that a large number of bacteria had not yet been developed in the naturally fermented sour porridge and artificially inoculated fermented sour porridge. In summary, there was little difference between the results of the macro-genome and the 16S rDNA sequencing at the genus level, while at the species level, more species were detected by the macro-genome sequencing.

We also analyzed the microbial communities of sour porridge at the species level. As shown in Supplementary Table S5, naturally fermented sour porridge contained a large number of unclassified *Bacillus*. Among the classified *Bacillus*, *Bacillus velezensis, Bacillus subtilis* and *Bacillus amyloliquefaciens* were the main species, accounting for 14.66, 4.42 and 2.85%, respectively. There were also small amounts of *S. harbinensis*, *Staphylococcus epidermidis*, *Lactobacillus paracasei*, etc. The *S. harbinensis* GX0002947-inoculated fermented sour porridge contained primarily *S. harbinensis*, accounting for 54.42%, followed by *Staphylococcus epidermidis*, accounting for 22.55%. There were also some other *Lactobacillus* species with lower contents, such as *Lactobacillus paracasei*, *Schleiferilactobacillus perolens*, *Lacticaseibacillus rhamnosus, Lapidilactobacillus bayanensis,* etc., totaling 32 species of *Lactobacillus*.

It could be seen that the microbial communities in naturally fermented sour porridge were rich, but there were also significant differences among samples, and there were certain uncertainties and risks. Since its fermentation depended on microbial communities naturally existing in the environment, the structure of the microbial communities in the fermented samples might change due to environmental conditions, resulting significant fluctuations in product quality. Compared with natural fermentation, the microbial community structure of artificially inoculated sour porridge is stable and controllable, allowing better control of fermentation conditions and improving the consistency and quality of product. In addition, artificially inoculated sour porridge can ensure the purity of the inoculated species during fermentation, reduce the impact of external contaminants on fermentation, and ensure product safety.

### 3.5 Metabolomics analysis of fermented sour porridge based on LC–MS

#### 3.5.1 OPLS-DA

OPLS-DA can provide an accurate measurement of the contribution of metabolites in the classification and identification of species. Consistent with the PCA results, OPLS-DA was also able to discriminate effectively between the samples, again indicating that there were significant differences in the metabolites of sour porridge samples between the two groups. As shown in Supplementary Figures S2, S3, R2 Y = 0.941, Q2 = 0.912 for the natural fermentation group ZR (comp 1), and R2 Y = 0.993, Q2 = 0.975 for the artificially inoculated fermentation group DD (comp 2), and the values of R2 Y and Q2 were close to 1. The results demonstrated the feasibility of the explanatory rate and predictive ability of the two models.

**Figure 7:**
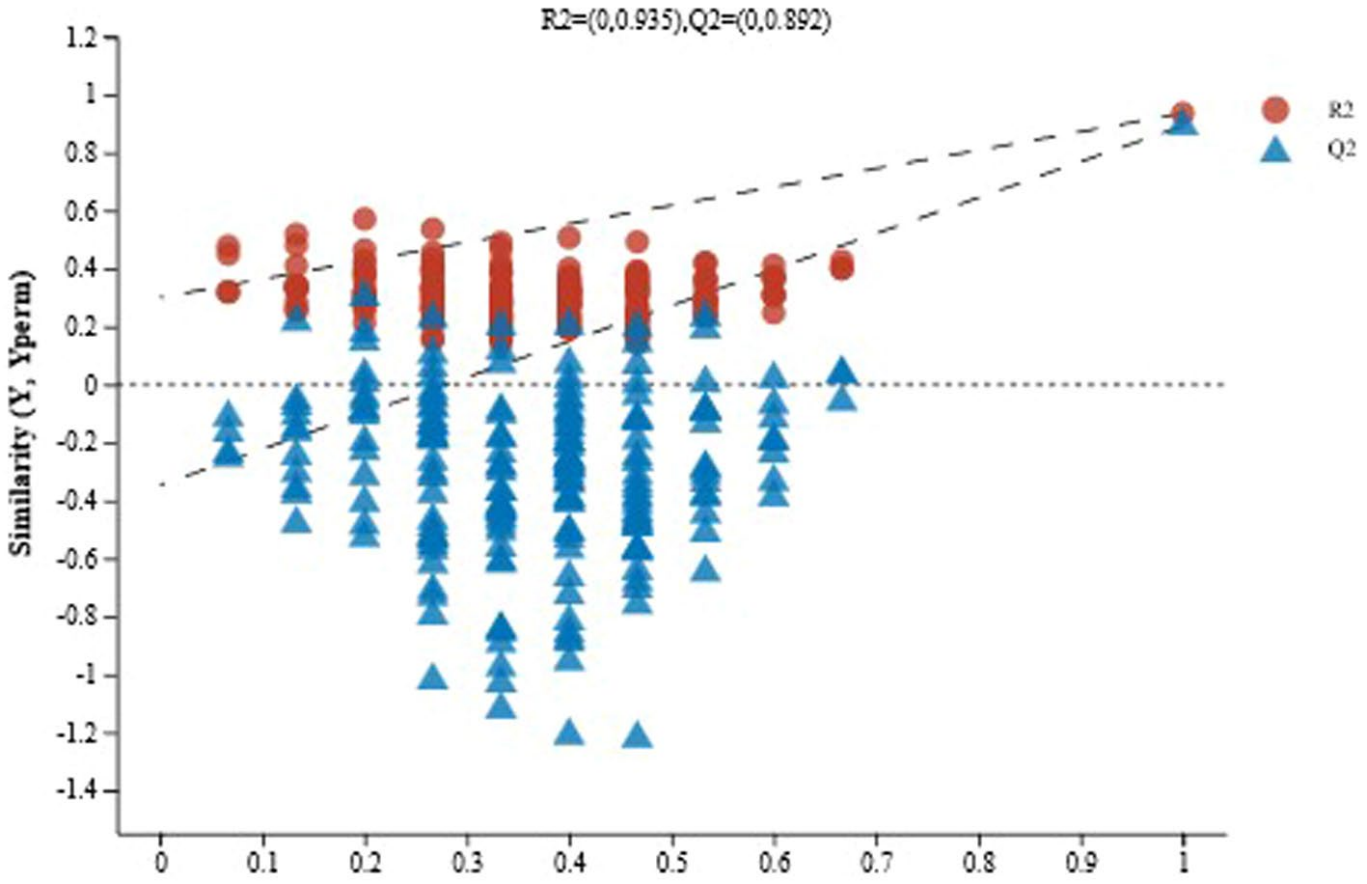
Displacement test chart of naturally fermented sour porridge and *S. harbinensis* GX0002947-inoculated fermented sour porridge.

The OPLS-DA method is prone to overfitting when sample sizes are small, producing false-positive results. To confirm this, it is necessary to perform replacement tests of the model. As shown in Figure 7, as the replacement retention decreased, R2 and Q2 showed a downward trend, while the regression line showed an upward trend, indicating that the replacement test was effective, and the model was reliable without overfitting.

#### 3.5.2 Types of metabolites and identification of differential metabolites

**Figure 8:**
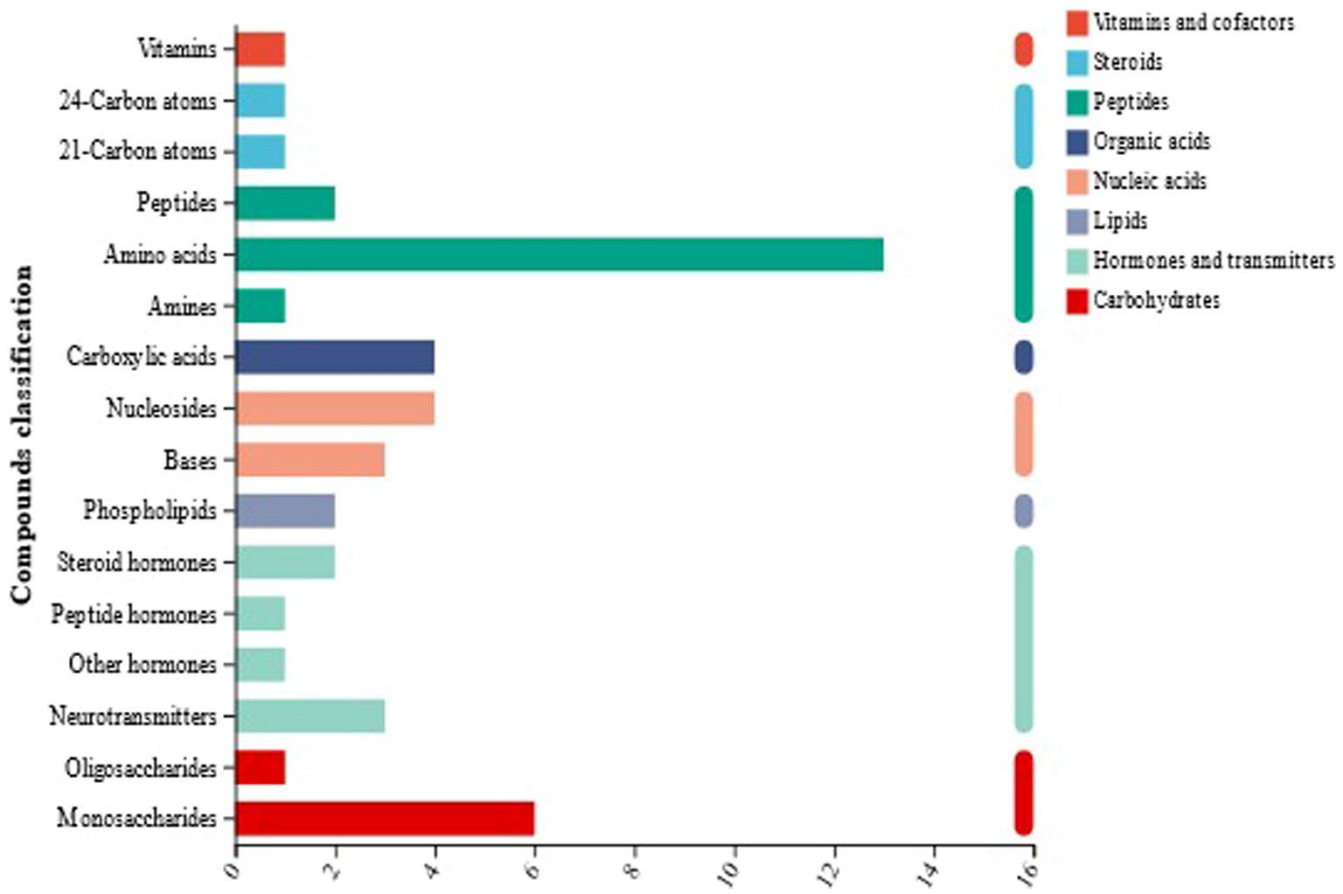
KEGG compound classification of naturally fermented sour porridge and *S. harbinensis* GX0002947-inoculated fermented sour porridge.

The metabolites identified by mass spectrometry were annotated using the KEGG database (Xu et al., 2022; Zhang et al., 2021; Wei et al., 2024). As shown in Figure 8, 96 metabolites were identified in the naturally fermented and artificially inoculated fermented sour porridge samples, of which peptides (mainly amino acids) accounted for 23.96%, lipids for 18.75%, carbohydrates for 11.46%, organic acids for 8.33%, and vitamins and antibiotics accounted for 5.21%. The classification and proportions of metabolites showed that organic acids, amino acids, carbohydrates, and lipids were the most abundant metabolites produced during sour porridge fermentation. Thus, the results showed that sour porridge was rich in organic acids, amino acids, and other nutrients after fermentation.

Based on the OPLS-DA results, 24 differential metabolites were identified between the two groups using the criteria of *p* < 0.05, VIP ≥ 1, and FC ≥ 1.5 conditions. As shown in Supplementary Table S6, the abundance of 17 metabolites was significantly increased while that of 7 metabolites was significantly decreased in the sour porridge fermented by *S. harbinensis* GX0002947 compared with the naturally fermented sour porridge samples.

As can be seen from Supplementary Table S6, the differential metabolites were primarily amino acids, carbohydrates, and lipids, as well as some other substances (functional substances such as flavoxate and elastin). The changes in the levels of these metabolites reflected the physiological and biochemical reactions occurring during the fermentation process. As organic compounds, amino acids are essential for the construction of proteins, but also are involved in the growth, reproduction, and metabolism of lactic acid bacteria (Zhao and Gänzle, 2018), and have important functions in the human body. The levels of various amino acids were increased in the artificially inoculated fermented sour porridge compared to naturally fermented sour porridge, which also explained the higher amino acid content in the *S. harbinensis* GX0002947-fermented sour porridge after the fermentation was completed. Among them, L-tryptophan is an essential amino acid and an important metabolic substance in the human body and cells. It is mainly involved in key metabolic pathways such as glycine, serine, and threonine metabolism, the biosynthesis of secondary metabolites for improving gut healthy (Zuo et al., 2025; Allison et al., 2018), mineral absorption, protein digestion and absorption, aminoacyl tRNA biosynthesis, and central carbon metabolism in cancer (Alessandra and Peter, 2021), reflecting its important roles in the growth and reproduction of lactic acid bacteria.

As a class of substances involved essentially in energy production, carbohydrates can be used to generate metabolic energy and microorganisms consume energy for their own growth and reproduction. Therefore, the metabolism of carbohydrates plays an important role in the survival and growth of lactic acid bacteria (Flint et al., 2012; Scott et al., 2013). Compared with the naturally fermented sour porridge, the levels of lactic acid in the artificially inoculated fermented sour porridge samples were significantly down-regulated. As one of the main metabolites produced by glycolysis, excessive accumulation of lactic acid can lead to acidification of the intracellular environment, lowering the cellular pH (Branson et al., 2022; Carpenter and Broadbent, 2009), and the acidic environment can also inhibit the activity of enzymes involved in glycolysis, thus inhibiting glycolysis. 4-Hydroxybutyric acid was found to be significantly up-regulated; this is mainly involved in key metabolic pathways such as carbon metabolism and the carbon fixation pathway in prokaryotes. Through the carbon fixation pathway, organic matter can be converted into inorganic matter, and the growth and reproduction of lactic acid bacteria can be promoted. This also fully explained why, compared with natural fermentation, the pH of the artificially inoculated fermented sour porridge dropped faster and the fermentation time was shorter. The results also explained the production of lactic acid, which could inhibit the growth of foodborne pathogenic bacteria. D-galactose was also found to be significantly up-regulated; this participates mainly in key metabolic pathways such as mineral absorption, digestion and absorption of carbohydrates, and sugar metabolism of amino acids and nucleotides. Lactose produces D-galactose under the action of lactic acid bacteria, which can enhance the absorption of minerals by bacteria, leading to improved growth and reproduction (Christelle et al., 2019).

Lipids are important components of cell membranes, important nutrients for the human body, and can also provide energy for the body. Compared with naturally fermented sour porridge, ganglioside GD2 (d18:1/18:1 (11Z)) and 10-Hydroxymelleolide were significantly up-regulated among the metabolites of artificially inoculated fermented sour porridge. Ganglioside GD2 has been found to be effective in treating neurological disorders, cancer immunotherapy, diagnosis of ovarian masses, etc. (Fleurence et al., 2017; Higashi et al., 2023; Levin et al., 2025). This also indicated that the fermented sour porridge might have nutritional health benefits. In addition to amino acids, carbohydrates and lipids, several differential metabolites belonging to other macromolecular groups were found, such as flavoxate, which is mainly involved in purine metabolism. As the most abundant metabolic substrates in living organisms, purines are involved in a variety of biological processes and can provide essential energy and cofactors. In addition, purine metabolism has also been associated with the regulation of tumors and cancers. The differential metabolites also included protein substances, such as elastin. This also explained to some extent the reason why the total protein content of the artificially inoculated fermented sour porridge was higher. In addition, this also has a positive effect on skin quality and the delay of aging (Lu et al., 2024).

## 4 Conclusion

In this study, we isolated and identified *S. harbinensis* GX0002947 from traditionally fermented sour porridge samples from Fusui County, Chongzuo City, Guangxi Zhuang Autonomous Region of China. Physiological and biochemical identification, tolerance analysis, and analysis of antibacterial properties showed that *S. harbinensis* GX0002947 met the conditions required for probiotics to exert probiotic activity, showed good resistance to both acid and bile salts, and was also effective in inhibiting the growth and reproduction of three foodborne pathogens, *Escherichia coli*, *Staphylococcus aureus*, and *Bacillus cereus*. The results showed that strain *S. harbinensis* GX0002947 was effective in the fermentation of cereals, and improved the nutritional value of cereals, such as increasing the content protein and amino acid. A total of 611 microbial species were detected by high-throughput sequencing of naturally fermented and artificially inoculated fermented sour porridge. The dominant phyla, genus and species of the naturally fermented sour porridge and artificially inoculated *S. harbinensis* GX0002947 fermented sour porridge are different. Comparative analysis showed that the community structure of the naturally fermented sour porridge was more complex and the fermented products were unstable, while the fermentation process of inoculated fermented sour porridge was controlled by the addition of specific strains, resulting in a more stable community structure and more consistent quality of the fermented product. A total of 24 differential metabolites were identified between the two groups of samples, and included amino acids, carbohydrates, and lipids. Sour porridge produces organic acids, amino acids, lipids, and carbohydrates through fermentation, enabling the conversion of macromolecules to small molecules, resulting in easier absorption by the human body. Compared with the naturally fermented sour porridge, the inoculated fermentation process resulted in a greater variety of functional small molecules, making the fermented sour porridge more nutritious and healthy, with probiotic functions, resulting in greater application prospects.

## Data Availability

The datasets presented in this study can be found in online repositories. The names of the repository/repositories and accession number(s) can be found in the article/Supplementary material.

## Footnotes

^1^ https://www.ezbiocloud.net/

^2^ www.i-sanger.com

## Supplementary material

SUPPLEMENTARY FIGURE S1 Morphological structure of *S. harbinensis* GX0002947. **(A)** Gram staining light microscope field of strain GX0002947 (1000×); **(B)** Electron microscope field of cell morphology of strain GX0002947 (10000×).

SUPPLEMENTARY FIGURE S2 Model evaluation parameter value of naturally fermented sour porridge and *S. harbinensis* GX0002947-inoculated fermented sour porridge.

SUPPLEMENTARY FIGURE S3 OPLS-DA model score diagram of naturally fermented sour porridge and *S. harbinensis* GX0002947-inoculated fermented sour porridge.
