# Supplementary figures and images for: Isolation and characterization of Schleiferilactobacillus harbinensis GX0002947 from naturally fermented sour porridge and its application in cereal fermentation
Source: Front Microbiol. 2025 Mar 31;16:1563733. doi: 10.3389/fmicb.2025.1563733 (PMC11994680; doi:10.3389/fmicb.2025.1563733)

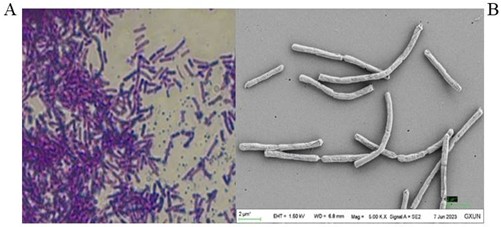

Supplement: SUPPLEMENTARY FIGURE S1 — Morphological structure of S. harbinensis GX0002947. (A) Gram staining light microscope field of strain GX0002947 (1000×); (B) Electron microscope field of cell morphology of strain GX0002947 (10000×). [file Image_1.JPEG]

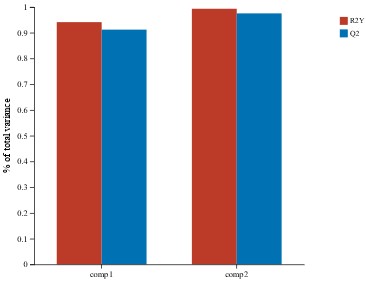

Supplement: SUPPLEMENTARY FIGURE S2 — Model evaluation parameter value of naturally fermented sour porridge and S. harbinensis GX0002947-inoculated fermented sour porridge. [file Image_2.JPEG]

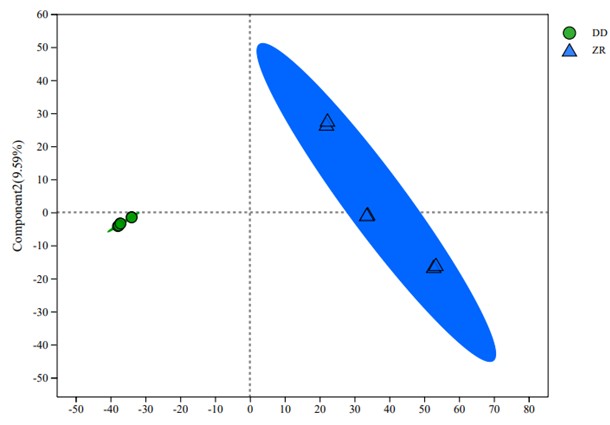

Supplement: SUPPLEMENTARY FIGURE S3 — OPLS-DA model score diagram of naturally fermented sour porridge and S. harbinensis GX0002947-inoculated fermented sour porridge. [file Image_3.JPEG]
