# Supplementary material for: Isolation and characterization of Schleiferilactobacillus harbinensis GX0002947 from naturally fermented sour porridge and its application in cereal fermentation
Source: Front Microbiol. 2025 Mar 31;16:1563733. doi: 10.3389/fmicb.2025.1563733 (PMC11994680; doi:10.3389/fmicb.2025.1563733)
Supplement: Supplementary file 4 [file Table_1.DOCX]

**TABLE S1** Utilization of sugar alcohols by *S. harbinensis* GX0002947 and *S. harbinensis* DSM16991.

| **Substrate** | **GX0002947** | **DSM16991** |
| --- | --- | --- |
| **Aesculin** | + | + |
| **Cellobiose** | + | + |
| **Maltose** | + | + |
| **Mannitol** | + | - |
| **Salicin** | + | - |
| **Sorbitol** | + | - |
| **Sucrose** | + | + |
| **Raffinose** | + | + |
| **Inulin** | + | + |
| **Lactose** | + | + |
| **1% Sodium hippurate** | + | - |

Note: “+”，Test positive，“-”, Test negative.
