# Supplementary material for: Isolation and characterization of Schleiferilactobacillus harbinensis GX0002947 from naturally fermented sour porridge and its application in cereal fermentation
Source: Front Microbiol. 2025 Mar 31;16:1563733. doi: 10.3389/fmicb.2025.1563733 (PMC11994680; doi:10.3389/fmicb.2025.1563733)
Supplement: Supplementary file 5 [file Table_2.DOCX]

**TABLE S2** Quantitative statistics of different status classification of naturally fermented sour porridge and *S. harbinensis* GX0002947 fermented sour porridge.

| **Samples** | **Phylum** | **Class** | **Order** | **Family** | **Genus** |
| --- | --- | --- | --- | --- | --- |
| **ZR-1** | 3 | 5 | 10 | 10 | 16 |
| **ZR-2** | 3 | 6 | 13 | 13 | 17 |
| **ZR-3** | 3 | 9 | 11 | 11 | 16 |
| **DD-1** | 3 | 8 | 9 | 12 | 11 |
| **DD-2** | 3 | 9 | 7 | 11 | 11 |
| **DD-3** | 3 | 9 | 7 | 11 | 11 |
| **Total** | 5 | 13 | 15 | 15 | 18 |
