# Supplementary material for: Isolation and characterization of Schleiferilactobacillus harbinensis GX0002947 from naturally fermented sour porridge and its application in cereal fermentation
Source: Front Microbiol. 2025 Mar 31;16:1563733. doi: 10.3389/fmicb.2025.1563733 (PMC11994680; doi:10.3389/fmicb.2025.1563733)
Supplement: Supplementary file 6 [file Table_3.DOCX]

**TABLE S3** Relative abundance of horizontal flora in phylum level of naturally fermented sour porridge and *S. harbinensis* GX0002947- inoculated fermented sour porridge.

| **Type of fermentation** | **Phylum** | **Level (%)** |
| --- | --- | --- |
| **Naturally fermented** | Firmicutes | 98.2 |
|  | Proteobacteria | 1.01 |
|  | Actinobacteria | 0.14 |
|  | Uroviricota | 0.09 |
|  | Streptophyta | 0.03 |
|  | Bacteroidota | 0.02 |
|  | Spirochaetes | 0.01 |
|  | Others | 0.5 |
| **Strain GX0002947 fermented** | Firmicutes | 98.0 |
|  | Uroviricota | 0.15 |
|  | Proteobacteria | 0.08 |
|  | Actinobacteria | 0.02 |
|  | unclassified_d__Bacteria | 1.75 |
