# Supplementary material for: Isolation and characterization of Schleiferilactobacillus harbinensis GX0002947 from naturally fermented sour porridge and its application in cereal fermentation
Source: Front Microbiol. 2025 Mar 31;16:1563733. doi: 10.3389/fmicb.2025.1563733 (PMC11994680; doi:10.3389/fmicb.2025.1563733)
Supplement: Supplementary file 7 [file Table_4.DOCX]

**TABLE S4** Relative abundance of horizontal flora in genus level of naturally fermented sour porridge and *S. harbinensis* GX0002947-inoculated fermented sour porridge.

| **Type of fermentation** | **Genus** | **Level (%)** |
| --- | --- | --- |
| **Naturally fermented** | *Bacillus* | 92.0 |
|  | *Paenibacillus* | 4.1 |
|  | *Staphylococcus* | 2.0 |
|  | *Priestia* | 0.7 |
|  | *Streptococcus* | 0.2 |
|  | Others | 1.0 |
| **Strain GX0002947 fermented** | *Schleiferilactobacillus* | 55.0 |
|  | *Staphylococcus* | 39.0 |
|  | *Bacillus* | 3.9 |
|  | *Lacticseibacillus* | 1.0 |
|  | *Lactobacillus* | 0.5 |
|  | Others | 0.6 |
