# Supplementary material for: Isolation and characterization of Schleiferilactobacillus harbinensis GX0002947 from naturally fermented sour porridge and its application in cereal fermentation
Source: Front Microbiol. 2025 Mar 31;16:1563733. doi: 10.3389/fmicb.2025.1563733 (PMC11994680; doi:10.3389/fmicb.2025.1563733)
Supplement: Supplementary file 8 [file Table_5.DOCX]

**TABLE S5** Relative abundance of horizontal flora in species level of naturally fermented sour porridge and *S. harbinensis* GX0002947-inoculated fermented sour porridge.

| **Type of fermentation** | **Species** | **Level (%)** |
| --- | --- | --- |
| **Naturally fermented** | *Unclassified Bacillus* | 73.89 |
|  | *Bacillus velezensis* | 14.66 |
|  | *Bacillus subtilis* | 4.42 |
|  | *Bacillus amyloliquefaciens* | 2.85 |
|  | *Schleiferilactobacillus harbinensis* | 0.25 |
|  | *Staphylococcus epidermidis* | 0.2 |
|  | Others | 3.73 |
| **Strain GX0002947 fermented** | *Schleiferilactobacillus harbinensis* | 54.42 |
|  | *Staphylococcus epidermidis* | 22.55 |
|  | *Unclassified Staphylococcus* | 13.99 |
|  | *Staphylococcus hominis* | 1.82 |
|  | *Lactobacillus* sp. | 1.08 |
|  | *Lactobacillus paracasei* | 0.85 |
|  | *Schleiferilactobacillus perolens* | 0.35 |
|  | *Lacticaseibacillus rhamnosus* | 0.19 |
|  | *Lapidilactobacillus bayanensis* | 0.11 |
|  | Others | 4.64 |
