# Supplementary material for: Isolation and characterization of Schleiferilactobacillus harbinensis GX0002947 from naturally fermented sour porridge and its application in cereal fermentation
Source: Front Microbiol. 2025 Mar 31;16:1563733. doi: 10.3389/fmicb.2025.1563733 (PMC11994680; doi:10.3389/fmicb.2025.1563733)
Supplement: Supplementary file 9 [file Table_6.DOCX]

**TABLE S6** Differential metabolites of naturally fermented sour porridge and *S. harbinensis* GX0002947-inoculated fermented sour porridge.

| **Form** | **Name** | **Molecular formula** | **Retention time（rt）** | **Mass-to-charge ratio（m/z）** | **Variations** |
| --- | --- | --- | --- | --- | --- |
| **Amino acid** | Cholylserine | C_27_H_45_NO_7_ | 2.6665 | 248.6657 | ↑ |
|  | N-Myristoyl Histidine | C_20_H_35_N_3_O_3_ | 0.6726 | 194.6314 | ↑ |
|  | Valyltyrosine | C_14_H_20_N_2_O_4_ | 2.7319 | 279.1353 | ↑ |
|  | Ala-phe | C_12_H_16_N_2_O_3_ | 3.0288 | 235.1086 | ↑ |
|  | Alanyltryptophan | C_14_H_17_N_3_O_3_ | 3.4442 | 274.1199 | ↑ |
|  | 5-Hydroxy-L-tryptophan | C_11_H_12_N_2_O_3_ | 1.8038 | 289.0989 | ↑ |
|  | L-Tryptophan | C_11_H_12_N_2_O_2_ | 3.0601 | 203.0821 | ↑ |
|  | Acetyl-Ser-Asp-Lys-Pro | C_20_H_33_N_5_O_9_ | 2.8173 | 532.2269 | ↓ |
|  | Isoleucyl-Methionine | C_11_H_22_N_2_O_35_ | 3.1157 | 261.1280 | ↑ |
|  | Gamma-Glu-leu | C_11_H_20_N_2_O_5_ | 2.8057 | 259.1300 | ↑ |
|  | Valylserine | C_8_H_16_N_2_O_4_ | 0.6758 | 203.1033 | ↑ |
| **Carbohydrate** | Raffinose | C_18_H_32_O_16_ | 0.6407 | 1009.3453 | ↑ |
|  | D-Galactose | C_6_H_12_O_6_ | 0.6088 | 198.0972 | ↑ |
|  | Palatinose | C_12_H_22_O_11_ | 0.6445 | 341.1090 | ↓ |
|  | Lactic Acid | C_3_H_6_O_3_ | 0.8960 | 269.0879 | ↓ |
|  | 4-Hydroxybutyric acid | C_4_H_8_O_3_ | 0.6407 | 247.0576 | ↑ |
| **Lipids** | Cyclopentolate | C_17_H_25_NO_3_ | 6.2482 | 581.3596 | ↓ |
|  | Ganglioside GD2 (d18:1/18:1(11Z)) | C_79_H_137_N_3_O_34_ | 5.3354 | 836.9602 | ↑ |
|  | N-Acetylmuramate | C_11_H_19_NO_8_ | 1.8038 | 276.1077 | ↓ |
|  | 10-Hydroxymelleolide | C_23_H_28_O_7_ | 6.5432 | 397.1689 | ↑ |
| **Others** | Flavoxate | C_24_H_25_NO_4_ | 3.3202 | 390.1676 | ↓ |
|  | Adenosine | C_10_H_13_N_5_O_4_ | 1.7958 | 268.1039 | ↓ |
|  | Indolelactic acid | C_11_H_9_NO_2_ | 3.0520 | 205.0970 | ↑ |
|  | Elastin | C_27_H_48_N_6_O_6_ | 2.7950 | 589.3115 | ↑ |

Note: Changes indicate a significant increase or decrease in the level of metabolites. ↑ means that compared with naturally fermented sour porridge, the differential metabolites are up-regulated; ↓ means that the differential metabolites are down-regulated.
